# Complete Genome Sequence of *Bacillus subtilis* Strain CU1050, Which Is Sensitive to Phage SPβ

**DOI:** 10.1128/genomeA.00262-16

**Published:** 2016-04-07

**Authors:** Christopher M. Johnson, Alan D. Grossman

**Affiliations:** Department of Biology, Massachusetts Institute of Technology, Cambridge, Massachusetts, USA

## Abstract

The Gram-positive bacterium *Bacillus subtilis* is used as a model organism to study cellular and molecular processes. Here, we announce the complete genomic sequence of *B. subtilis* strain CU1050, derived from *B. subtilis* strain 168. CU1050 has historically been used to study suppressor mutations and phage biology, especially the lysogenic phage SPβ.

## GENOME ANNOUNCEMENT

*Bacillus subtilis* has been used to study many fundamental biological processes, including sporulation, competence development, and horizontal gene transfer (1). The temperate phage SPβ is found in many common laboratory strains of *B. subtilis*, including strain 168. The study of SPβ was greatly facilitated by the identification of a sensitive strain, *B. subtilis* CU1050 (originally *su + 3*) (2–8).

We report here the genome sequence of CU1050. This strain is derived from 168 and was mutagenized using 2-aminopurine to generate a nonsense suppressor (9). The suppressor mutation is a T-to-A transversion that changes the anticodon of the gene encoding tRNA-lys in the *trnS* operon from UUU to UUA (10), causing lysine to be inserted at ochre nonsense mutations (11). The strain also supports efficient plaque formation by certain defective mutants of øe (9), the temperate phage SPβ, for which CU1050 is null (2), and the temperate phage H2 from *Bacillus amyloliquefaciens* (12).

CU1050 has a circular chromosome of 4,056,281 bp. We prepared genomic DNA by phenol-chloroform extraction with RNase A treatment, followed by ethanol precipitation. We sheared DNA using a Covaris sonicator, recovered 300- to 600-bp fragments using a Beckman Coulter SPRIworks system, and obtained 150-bp paired-end reads using an Illumina MiSeq. A total of 1,488,671 reads assembled to the final genome, giving 55-fold mean coverage. We used *de novo* assembly with Velvet (13) and whole-genome alignment with Mauve (14) to identify potential large differences between CU1050 and strain 168 (accession no. NC_000964). We used breseq (15) to identify and correct polymorphisms, using 2 rounds of refinement, and then used Geneious (Biomatters) to compare the reads to the resulting sequence for a further round of refinement. Gene prediction was done using the NCBI Prokaryotic Genome Annotation Pipeline (16).

Compared to strain 168 (17, 18), strain CU1050 is null for the mobile genetic elements SPβ and ICE*Bs1*, has a 4,123-bp deletion in the region from *yozF* to *yoaU*, a 255-bp deletion of the intergenic region from *ydbT* to *ydcA*, a 45-bp in-frame deletion within *codY*, and other smaller deletions. It also contains approximately 3,950 single-nucleotide polymorphisms (SNPs) and indels, most of which are located in discrete regions, including the *panB* to *hepT* hypervariable region (30 kb) (19) and other smaller regions: *ydbM* to *ydcC* (12 kb), *pbuE* to *rrnE* (11.5 kb), *yqfU* to *sigA* (10 kb), *moaB* to *ackA* (2 kb), and *sacA* to *ywcL* (5 kb). We confirmed that CU1050 carries nonsense mutations in *metA* and *thrC* and the previously identified nonsense suppressor mutation.

Different auxotrophic requirements have been reported for CU1050 (3, 9). We found that CU1050 requires supplementation with methionine, threonine, and leucine, but not adenine, when grown in S7_50_ defined minimal medium (20). Suppression of the *metA* and *thrC* mutations is evidently not sufficient to support normal growth in this medium lacking methionine and threonine.

### Nucleotide sequence accession number.

The complete genome sequence is available from GenBank under accession no. CP014166. Strain CU1050 is available from the Bacillus Genetic Stock Center.
